# Supplementary material for: Trichosporon asahii PLA2 Gene Enhances Drug Resistance to Azoles by Improving Drug Efflux and Biofilm Formation
Source: Int J Mol Sci. 2023 May 16;24(10):8855. doi: 10.3390/ijms24108855 (PMC10219205; doi:10.3390/ijms24108855)
Supplement: Supplementary file 1 [file ijms-24-08855-s001.zip › ijms-2356426-supplementary.pdf]

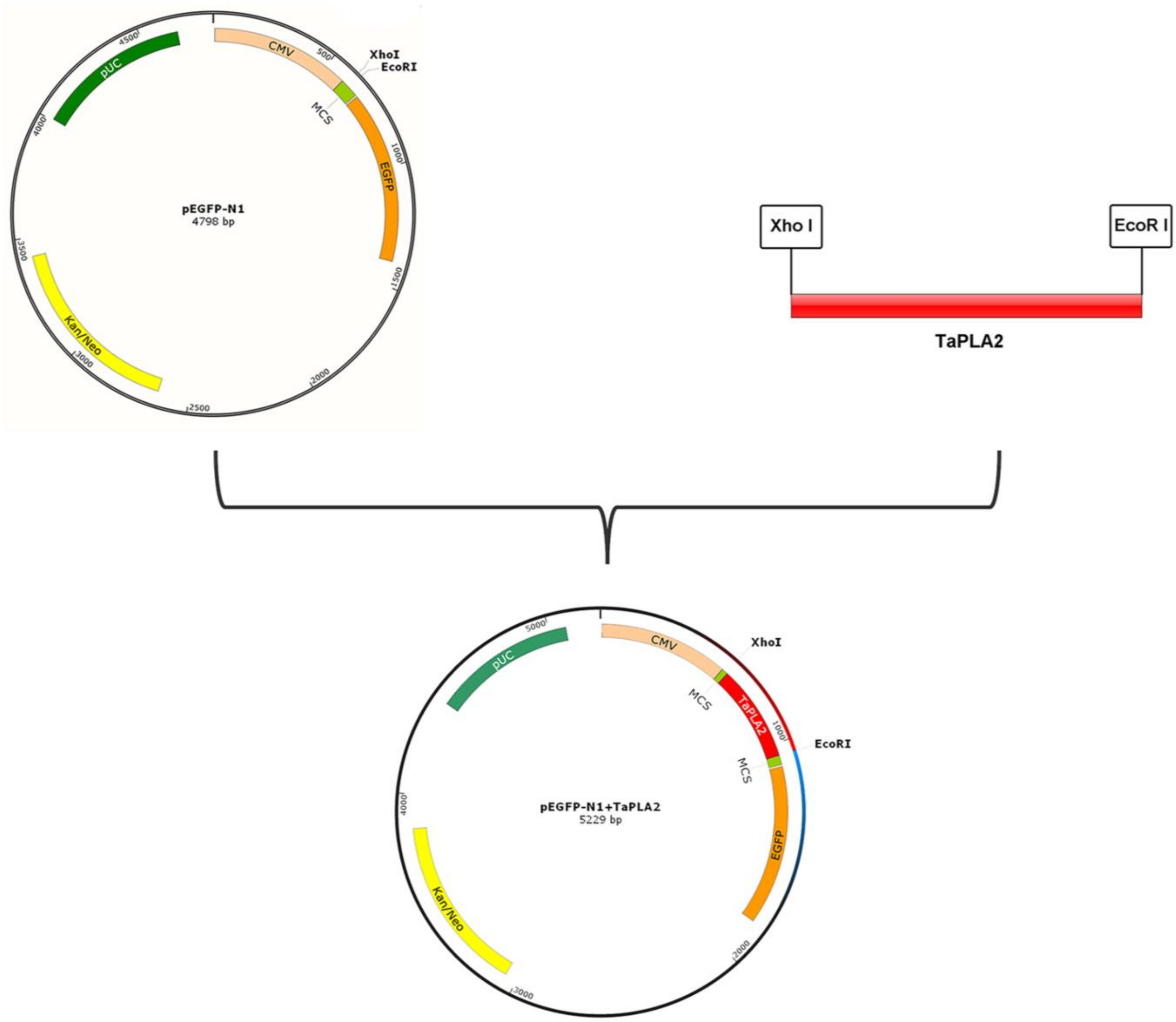

Figure S1. Construction of technical route used for *T. asahii* recombinant plasmids. The pEGFP-N1-TaPLA2 plasmid was used to construct the overexpressed TaPLA2 gene strain.

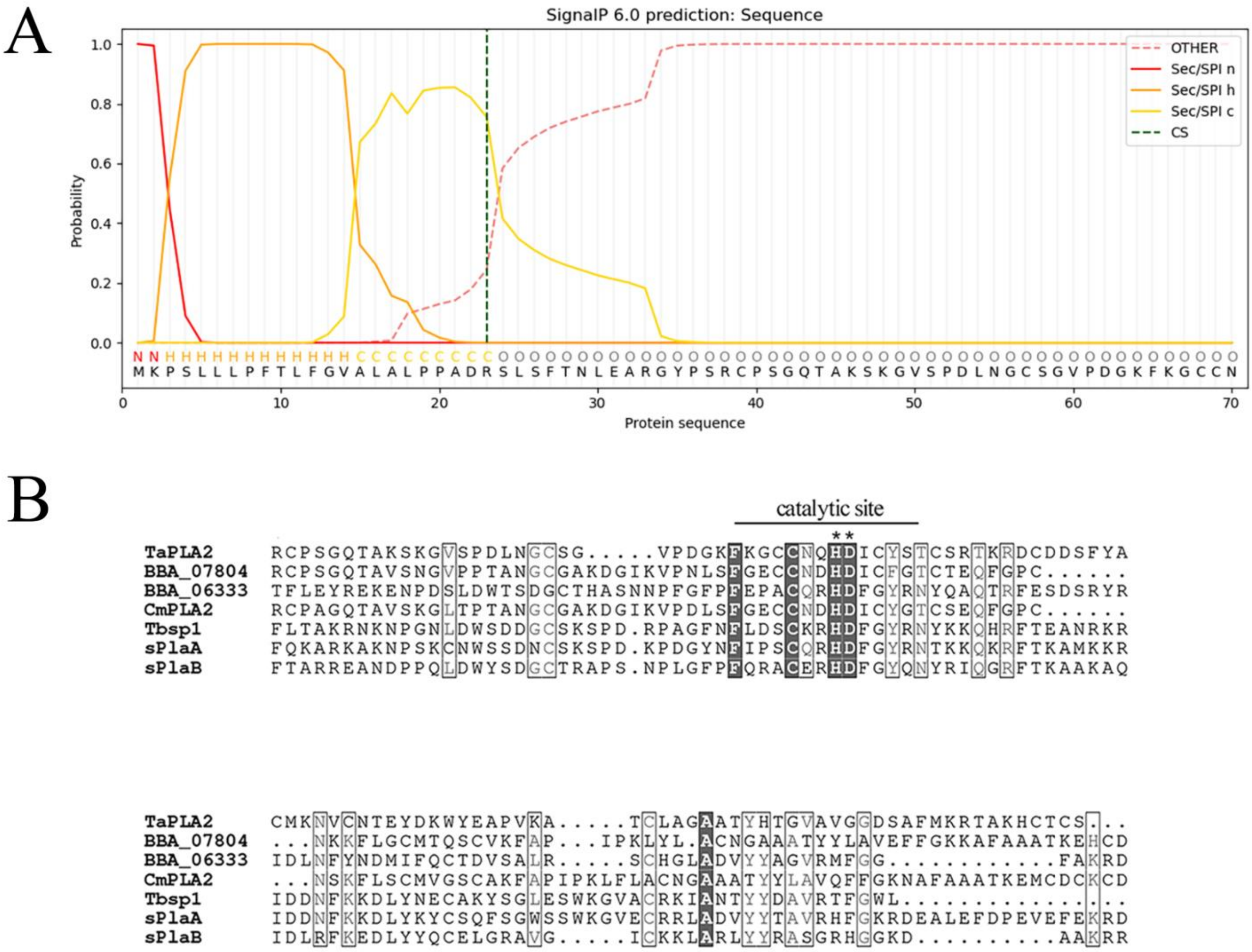

Figure S2. (**A**) Signal peptide of TaPLA2, which was predicted to contain a signal peptide at positions 1 to 23, with the signal peptide sequence MKPSLLLPFTLFGVALALPPADR. (**B**) Alignment of the conserved domains of PLA2 amino acid sequences from different fungal species, as indicated, which contains the typical Ca^{2+} binding and catalytic site C-C-x-x-H-x-C-C motif, the conserved His/Asp dimer (HD), and a potential disulfide bond.

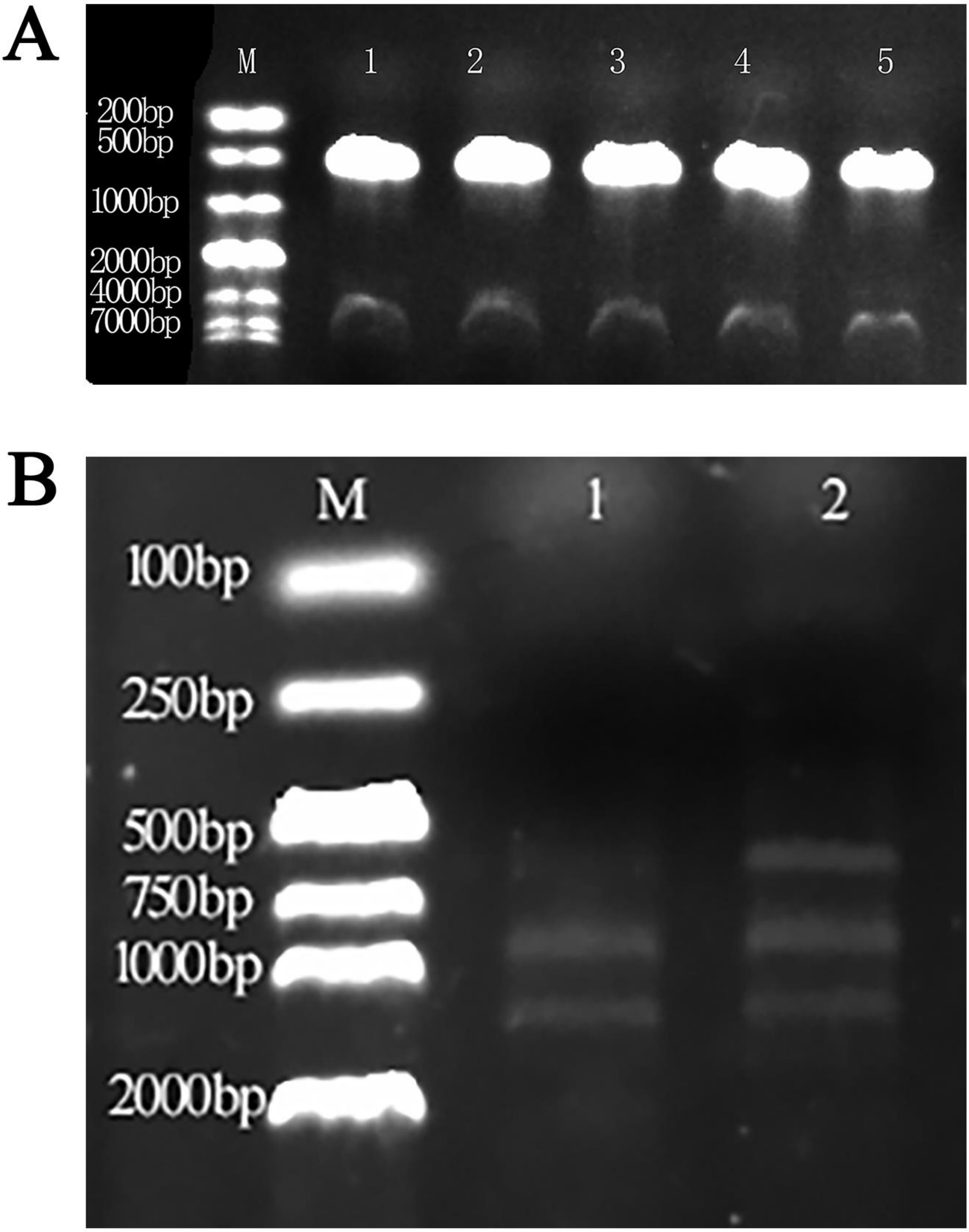

Figure S3. (**A**) PCR verification of pEGFP-N1-TaPLA2 recombinant plasmid. M is the DL10000 DNA marker. Lanes 1–5 show PCR production of the TaPLA2 gene (approximately 500 bp) and the liner plasmid (approximately 4000 bp). (**B**) EGFP tag verified by PCR. M is the DL2000 DNA marker; Lanes 1 and 2 show results for YAN0802 and the TaPLA2^{OE} mutant, respectively.

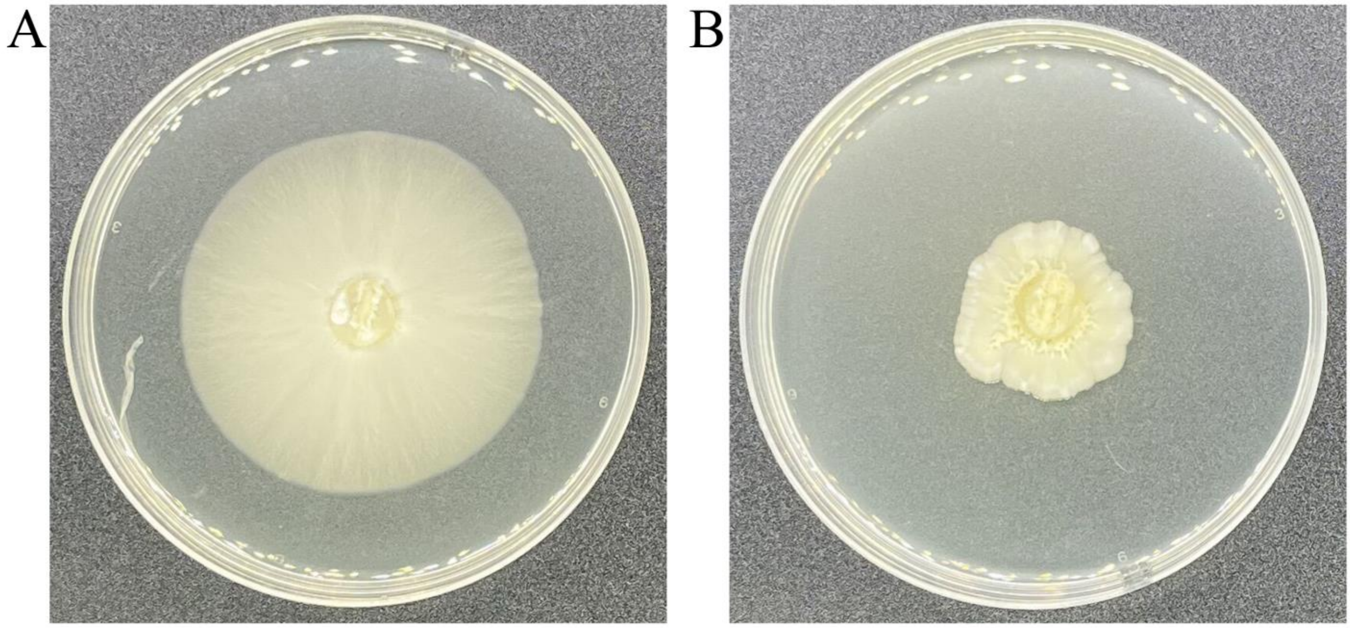

Figure S4. Growth phenotypes of wild strain YAN0802 and overexpressed mutant TaPLA2^{OE}. (**A**) YAN0802 colony is large, transparent, and round with long mycelium. (**B**) TaPLA2^{OE} colony is opaque with irregular edges and short hypha.

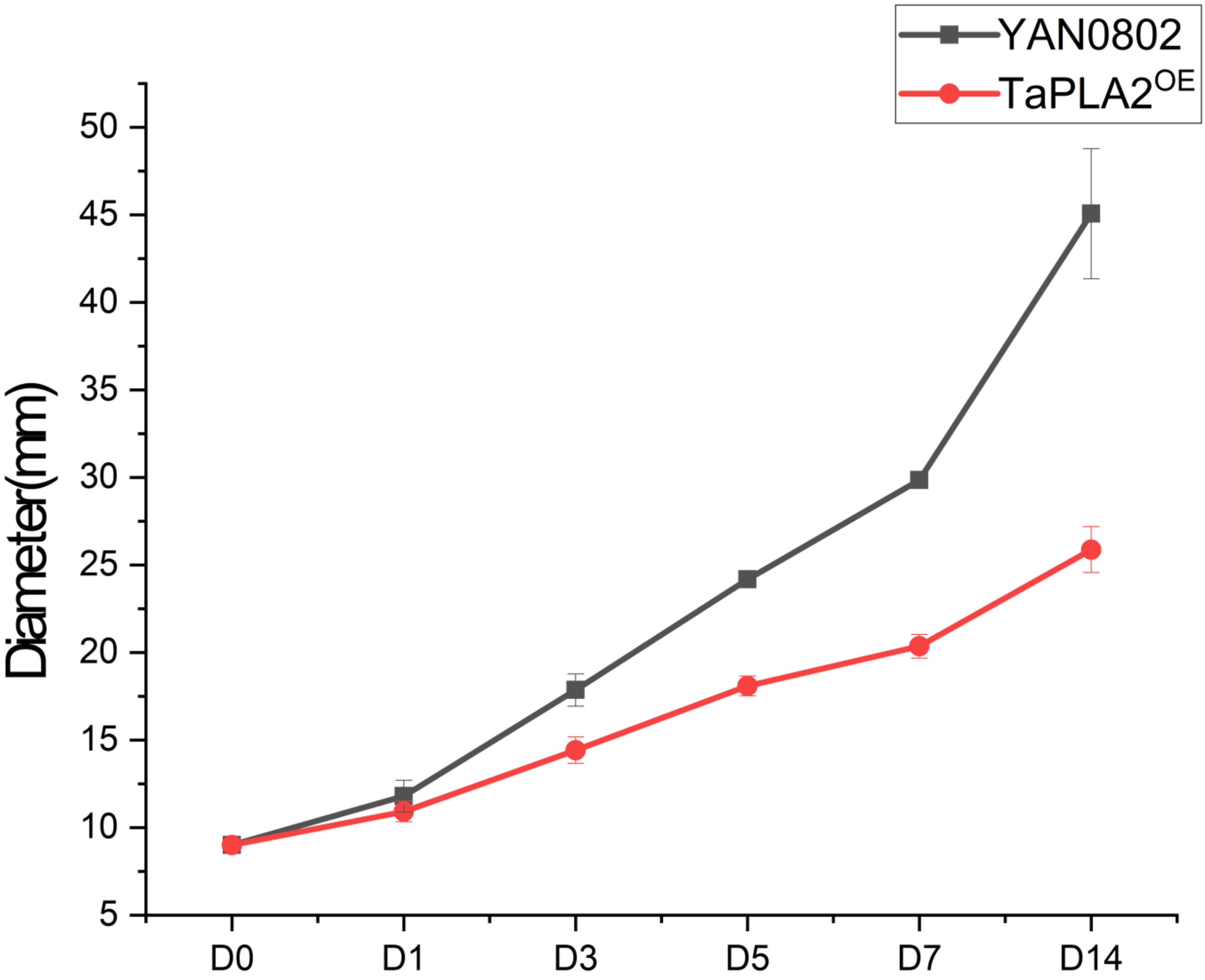

Figure S5. The growth curves of wild YAN0802 and TaPLA2^{OE} mutant.

Table S1. Primers used to construct the overexpressed strain.

**Primers**	**Nucleic Acid Sequence (5' to 3')**
TaPLA2 for cloning	
PLA2	F: CGGCAACAGTTCACCATGAAGCCCT
	R: GCATGCCCACCTAGGAGCAGGTACA
Primers were linked by homology arms	
TY	F: CTACCGGACTCAGAT CTCGAG ATGAAGCCCTCCCTTCTCCTCCCCT
	R: GTACCGTCGACTGCA GAATTC CTAGGAGCAGGTACAGTGCTTCGCG
Validation of recombinant plasmids	
CMV-F	CGCAAATGGGCGGTAGGCGTG
PLA2-R	GCATGCCCACCTAGGAGCAGGTACA
Primers for EGFP amplification	
EGFP	F: CTGGTCGAGCTGGACGGCGACG
	R: CACGAACTCCAGCAGGACCATG
Primers for RT-PCR	
YG	F: GGCAAGTTCAAAGGCTGCTG
	R: CACATTCTTCATGCACGCGT
18s rRNA	F: TCTTTCTTGATTTTGTGGGTGG
	R: TCGATAGTCCCTCTAAGAAGTG

Table S2. Primers for genes involved in efflux pumps.

Primers	Nucleic Acid Sequence (5' to 3')
Primers for ATP-binding cassette (ABC) Transporters	
CDR4	F: ACGACCTCAGGCCTGTATCT
	R: GGCGAGGATTACGGTACTGG
PDR11p	F: TGCAGGCCTTCTTCATGGAG
	R: CCAGACCAGGTGAAGGGAAC
ABC-T	F: CAAGGTCGGATTGGAGGGAC
	R: TGATGTCTGCGCCATCCTTT
CDR1	F: TTTGACGATGTGGGGAGTGG
	R: GTACGCAATGACGAAACCGG
Primers for Major Facilitator Superfamily (MFS) transporters	
FUB11	F: GCCGGTGTTTTTGGCTGTAG
	R: GCATGGGCTCATACGTCTCA
MTP	F: CCGTTTCCTCGCTGGATTCT
	R: CGAAGATGGCAATGGCGAAG
MDR	F: CTTCTCTGGGCTCCTGCTTC
	R: AGAGGGGACGATCCGAAGAA

Table S3. Primers for genes involved in cell-wall integrity.

Primers	Nucleic Acid Sequence (5' to 3')
Chitin synthesis or degradation of gene primers	
CHSe	F: CTACCTCGTGTACCCCCTCA
	R: TACGACGTAGAAGCCGATGC
CHS1	F: ACCAGTTCTACCACACGCAG
	R: ACGAAACATCCTGGCGTCAT
CHS3	F: TGTACTCGCTCGACTCTGGA
	R: TCATTGGCGTGAATCCCGAA
CHS5	F: ATCCAACCACCAACGCTCAT
	R: GACGAGCAAACAAAGTGCGT
CHT1	F: CCTACCCCGACAACAAGGAC
	R: AATGTAGAAGTGGGTGGCGG
CHT2	F: GTGGACTCACGACACTGAGG
	R: GAGGATGAGAGCAGGCTTGG
CHT3	F: TCCCCAGGGCTTTGTGATTC
	R: CCAGTCGATCTCGCTTACCC
Cell wall synthesis-related primers	
GPI	F: CGGATATTGCGGATGGGCT
	R: CCAGACGACTTGGGCTTGT
ALS	F: TCAGACAAGATGCACCGTCC
	R: TGAGGAGGTGACGCTTGAAC
HOG-MAPK signaling pathway primer	
HOG1	F: CTCGTCCGAATAGCCCTGAC
	R: TCGTGACGGAGGTGCTTTAG
MAPK	F: CATTCACTCTGCGAACGTGC
	R: CTGCTCAGTCTGTACCTGCC
